# Elevated CO_2_ Concentration Alters Photosynthetic Performances under Fluctuating Light in *Arabidopsis thaliana*

**DOI:** 10.3390/cells10092329

**Published:** 2021-09-06

**Authors:** Shun-Ling Tan, Xing Huang, Wei-Qi Li, Shi-Bao Zhang, Wei Huang

**Affiliations:** 1 Kunming Institute of Botany, Chinese Academy of Sciences, Kunming 650201, China; tanshunling@mail.kib.ac.cn (S.-L.T.); huangxing@mail.kib.ac.cn (X.H.); weiqili@mail.kib.ac.cn (W.-Q.L.); 2 University of Chinese Academy of Sciences, Beijing 100049, China

**Keywords:** CO_2_ concentration, photosynthesis, photosystem I, redox state of P700

## Abstract

In view of the current and expected future rise in atmospheric CO_2_ concentrations, we examined the effect of elevated CO_2_ on photoinhibition of photosystem I (PSI) under fluctuating light in *Arabidopsis thaliana*. At 400 ppm CO_2_, PSI showed a transient over-reduction within the first 30 s after transition from dark to actinic light. Under the same CO_2_ conditions, PSI was highly reduced after a transition from low to high light for 20 s. However, such PSI over-reduction greatly decreased when measured in 800 ppm CO_2_, indicating that elevated atmospheric CO_2_ facilitates the rapid oxidation of PSI under fluctuating light. Furthermore, after fluctuating light treatment, residual PSI activity was significantly higher in 800 ppm CO_2_ than in 400 ppm CO_2_, suggesting that elevated atmospheric CO_2_ mitigates PSI photoinhibition under fluctuating light. We further demonstrate that elevated CO_2_ does not affect PSI activity under fluctuating light via changes in non-photochemical quenching or cyclic electron transport, but rather from a rapid electron sink driven by CO_2_ fixation. Therefore, elevated CO_2_ mitigates PSI photoinhibition under fluctuating light at the acceptor rather than the donor side. Taken together, these observations indicate that elevated atmospheric CO_2_ can have large effects on thylakoid reactions under fluctuating light.

## 1. Introduction

Photosynthetic organisms absorb light energy to drive photosynthetic electron flow and CO_2_ assimilation. In linear electron flow (LEF), electrons are transferred from photosystem II (PSII) to photosystem I (PSI), and ultimately to NADP^+^, producing NADPH. This electron flow is coupled to the formation of proton motive force that powers the regeneration of ATP. In cyclic electron flow (CEF) around PSI, electrons are transported from ferredoxin into the plastoquinone pool, generating ATP without producing NADPH. PSI and PSII work co-operatively to form ATP and NADPH, which is essential for the primary metabolism. Once PSII is photoinhibited, as indicated by the decrease in the maximum quantum yield of PSII (*F_v_*/*F_m_*), LEF would be suppressed [1]. Once PSI is photodamaged, as indicated by the decrease in the maximum photo-oxidizable P700 (*P_m_*), both LEF and CEF are depressed, which will affect CO_2_ fixation and impair plant growth [2,3,4,5,6].

Photosynthetic organisms are often exposed to dynamic fluctuations in light intensity when grown in the field [7,8,9]. Under such fluctuating light (FL) conditions, an abrupt increase in light intensity will lead to an immediate rise in light absorption, triggering electron flow from PSII to PSI [10,11,12]. Meanwhile, CO_2_ assimilation has a much slower kinetics than electron flow from PSII [7,13,14]. Therefore, NADPH cannot be immediately consumed by CO_2_ assimilation. Because the pool size of NADPH is relatively small, such an imbalance between electron flow and primary metabolism leads to an increase in the NADPH/NADP^+^ ratio. The lack of NADP^+^ restricts LEF and thus induces the accumulation of excited states at PSI, resulting in the generation of reactive oxygen species (ROS) in PSI [15,16]. Because the ROS produced in PSI cannot be immediately scavenged by antioxidant system [17], FL can cause selective photodamage to PSI [16,18,19].

Accordingly, photosynthetic organisms employ several alternative electron transport routes to protect PSI under FL [20,21,22]. In Arabidopsis (*A. thaliana*), the *proton gradient regulation 5* (*pgr5*) is seedling-lethal when grown under FL, as a consequence of uncontrolled PSI photoinhibition due to a defect in CEF [23]. Therefore, CEF is essential for PSI photoprotection under FL in angiosperms [16,24,25]. During CEF, electrons from ferredoxin are transferred to plastoquinone, generating a ΔpH without reducing NADP^+^ [24,26,27,28]. In response to a sudden increase in irradiance, CEF first rises before gradually decreasing and reaching a constant rate [29,30,31,32]. The initial stimulation of CEF facilitates the rapid formation of ΔpH [29], slowing down the electron flow at the cytochrome (Cyt) *b_6_*/*f* complex and increasing the ATP/NADPH production ratio [33,34]. CEF therefore protects PSI under FL at both electron donor and acceptor sides [16]. Once ΔpH reaches a sufficient level, CEF activity decreases to a steady state to avoid over-acidification of the thylakoid lumen, thus optimizing the tradeoff between photosynthesis and photoprotection [29,35]. Therefore, CEF plasticity plays an important role in sustaining photosynthesis under FL.

One of the main drivers of global climate change, atmospheric CO_2_ concentrations are expected to continue increasing in the near future. Higher atmospheric CO_2_ may lead to a rise in intercellular and chloroplast CO_2_ concentrations, which will boost the rate of CO_2_ assimilation and plant growth [36,37]. However, the maintenance of a high level of PSI activity is essential for optimal photosynthesis. Once PSI photoinhibition occurs under FL, the rate of CO_2_ assimilation under higher atmospheric CO_2_ conditions will also suffer [3]. Therefore, the predicted positive effects of elevated atmospheric CO_2_ on crop yield are not only a reflection of the rate of CO_2_ fixation, but are also likely linked to light reactions. However, how elevated atmospheric CO_2_ affects light reactions under FL is largely known. 

Theoretically, an elevated concentration of atmospheric CO_2_ will raise the photosynthetic induction rate when transitioning to high light [38,39], increasing the NADPH consumption rate and leading to an increase in the NADP^+^/NADPH ratio. Consequently, elevated atmospheric CO_2_ concentrations might facilitate electron flow from PSI to NADP^+^, with the potential to alleviate the over-reduction of PSI under FL. However, it is unclear whether elevated atmospheric CO_2_ will in fact break the imbalance between light and dark reactions of photosynthesis and alleviate PSI photoinhibition under FL. In the present study, we measured the chlorophyll fluorescence and PSI signals under FL at 400 and 800 ppm CO_2_ concentrations in Arabidopsis leaves. The aims of this study were to (1) assess the effect of elevated CO_2_ on the redox state of PSI under FL and (2) examine whether elevated CO_2_ can mitigate PSI photoinhibition under FL.

## 2. Materials and Methods

### 2.1. Plant Materials

Arabidopsis (*Arabidopsis thaliana*) wild-type plants grown in a greenhouse (light intensity~100 μmol photons m^−2^ s^−1^, 12-h photoperiod, 25 °C, 60% humidity, 400 ppm CO_2_) for 6–8 weeks after germination. We used fully expanded but not senescent leaves for photosynthetic measurements.

### 2.2. Measurement of P700 Redox Kinetics

After incubation in darkness for 60 min, we used a Dual-PAM 100 measuring system (Heinz Walz, Effeltrich, Germany) to record the P700 redox kinetics following the transition from darkness to a light intensity of 1809 μmol photons m^−2^ s^−1^ for 20 s at 400 or 800 ppm CO_2_. All photosynthetic measurements were conducted in a phytotron. The air temperature was set to 25 °C; the relative humidity was set to 60%; the CO_2_ concentrations were set to 60% and 400 or 800 ppm. 

### 2.3. PSI and PSII Measurements

After dark adaptation for 15 min, plants were illuminated at 272 μmol photons m^−2^ s^−1^ for 10 min to activate photosynthesis. Afterward, plants were exposed to FL alternating between LL (59 μmol photons m^−2^ s^−1^) and HL (1809 μmol photons m^−2^ s^−1^), and the changes in PSI and PSII parameters were recorded using a Dual-PAM 100 measuring system. The quantum yield of PSI photochemistry (Y(I)), the quantum yield of PSI non-photochemical energy dissipation due to donor side limitation (Y(ND)), and the quantum yield of non-photochemical energy dissipation due to acceptor side limitation (Y(NA)) were calculated with the following formulas [40]: Y(I) = (*P_m_′* − *P*)/*P_m_*, Y(ND) = *P*/*P_m_* and Y(NA) = (*P_m_* − *P_m_′*)/*P_m_*.

The PSII parameters were calculated with another three formulas [41,42]: Y(II) = (*F_m_′* − *F_s_*)/*F_m_′*, Y(NO) = *F_s_*/*F_m_* and NPQ = (*F_m_* − *F_m_′*)/*F_m_′*. Y(II) was the effective quantum yield; Y(NO) was the quantum yield of non-regulated energy dissipation in PSII; NPQ was the non-photochemical quenching in PSII. *F_s_* was the steady state after light adaptation. *F_m_* and *F_m_′* represented the maximum fluorescence after dark and light adaptation, respectively. *F_m_* was measured after dark-adaptation for 15 min. The photosynthetic electron transport rate through PSI (or PSII) was calculated as: ETRI (or ETRII) = PPFD × Y(I) (or Y(II)) × 0.84 × 0.5, where PPFD is the photosynthetic photon flux density, and the light absorption of incident irradiance is assumed to be 0.84.

### 2.4. Statistical Analysis

All results are displayed as mean values of five individual experiments. *t*-tests were used to determine the significant differences between different treatments (*α* = 0.05).

## 3. Results

### 3.1. Elevated Atmospheric CO_2_ Affects the PSI Redox State after Transition from Dark to Light

**Figure 1:**
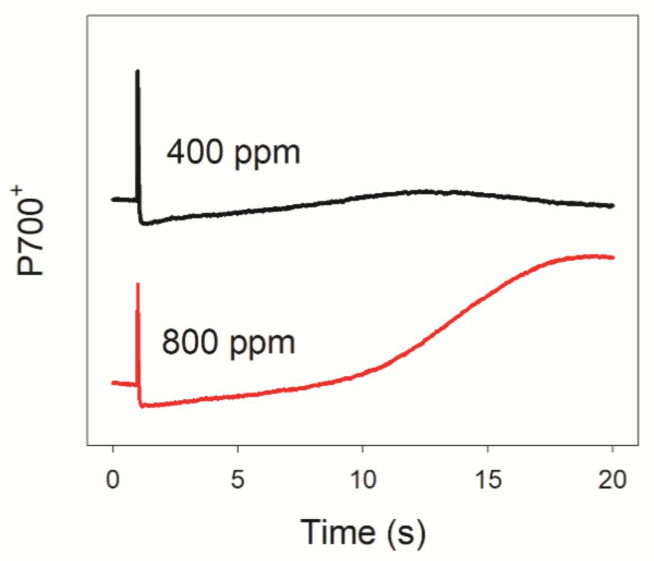
Changes in P700 redox kinetics after transition from darkness to actinic light (1809 μmol photons m^−2^ s^−1^) in 400 and 800 ppm atmospheric CO_2_. Data are shown as mean values of five leaves from five individual plants.

After transition from darkness to 1809 μmol photons m^−2^ s^−1^, P700 became gradually re-oxidized in 20 s when measured in 800 ppm CO_2_ (Figure 1). However, we failed to observe a similar re-oxidation of P700 in 400 ppm CO_2_ (Figure 1). The rapid re-oxidation of P700 after transition from darkness to light is attributed to the outflow of electrons from PSI to downstream electron acceptors [43,44,45]. Because photo-reduction of O_2_ mediated by flavodiiron proteins and water–water cycle are not observed in *A. thaliana*, the difference in P700 redox kinetics between 400 and 800 ppm CO_2_ suggested that elevated atmospheric CO_2_ accelerates the outflow of electrons from PSI to NADP^+^, probably due to the increased rate of CO_2_ fixation.

**Figure 2:**
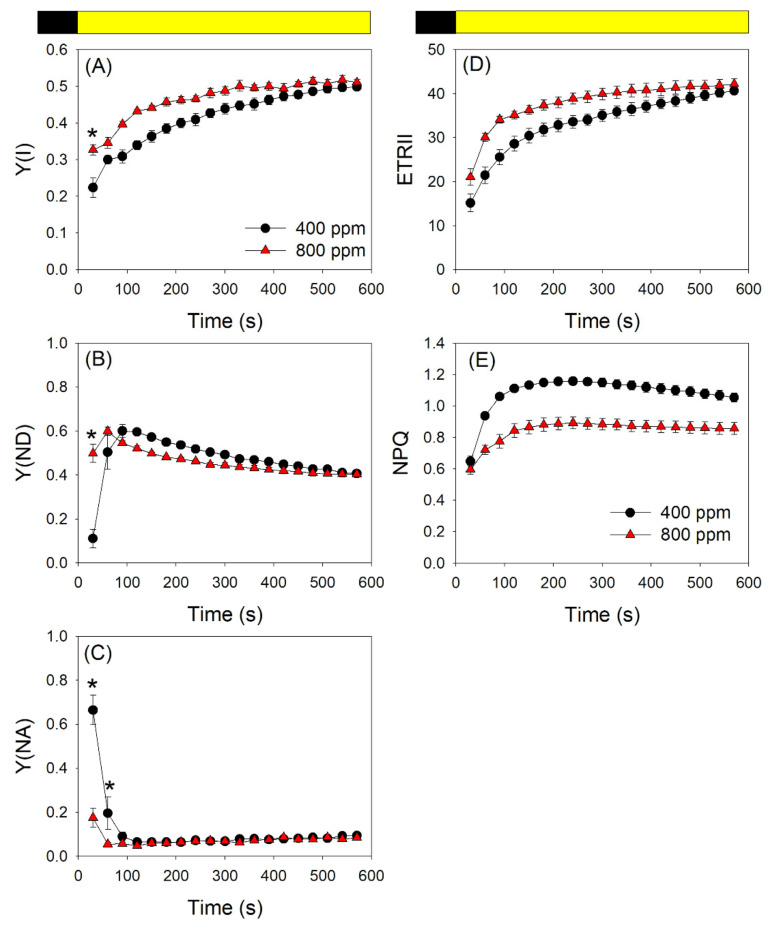
Changes in PSI and PSII parameters after transition from darkness to 272 μmol photons m^−2^ s^−1^ in Arabidopsis leaves, measured in 400 and 800 ppm CO_2_. (**A**) Y(I), the quantum yield of PSI photochemistry; (**B**) Y(ND), the quantum yield of PSI non-photochemical energy dissipation due to the donor side limitation; (**C**) Y(NA), the quantum yield of PSI non-photochemical energy dissipation due to the acceptor side limitation; (**D**) ETRII, electron transport rate through PSII; (**E**) NPQ, non-photochemical quenching in PSII. Data are shown as means ± SD (*n* = 5). Asterisk indicates a significant different between 400 and 800 ppm.

During photosynthetic induction at a moderate light of 272 μmol photons m^−2^ s^−1^, the quantum yield of PSI photochemistry (Y(I)) was enhanced at 800 ppm CO_2_ (Figure 2A). The quantum yield of PSI non-photochemical energy dissipation due to the donor side limitation (Y(ND)) remained at a low level for 30 s upon transfer from dark to light when measured in 400 ppm CO_2_ (Figure 2B). This led to the over-reduction of PSI, as indicated by the high value of PSI acceptor side limitation (Y(NA)) at the same time point (Figure 2C). By comparison, Y(ND) had already almost reached its maximal value within 30 s in 800 ppm CO_2_ (Figure 2B), resulting in a correspondingly low Y(NA) value (Figure 2C). Therefore, elevated atmospheric CO_2_ concentrations significantly affected the redox state of PSI during transition from darkness to actinic light. By contrast, elevated atmospheric CO_2_ had minor effects on electron flow from PSII (ETRII) and non-photochemical quenching (NPQ) within the first 30 s after transition from darkness to light (Figure 2D,E). Thus, we concluded that the effects of elevated CO_2_ concentration on PSI redox state are not caused by electron flow from PSII or the formation of a ΔpH. Instead, an increase in CO_2_ concentration raises the rate of CO_2_ fixation and thus facilitates electron transfer from PSI to NADP^+^, which in turn alleviates the over-reduction of PSI.

### 3.2. Elevated Atmospheric CO_2_ Affect PSI and PSII Performances Differently after Transition from LL to HL

**Figure 3:**
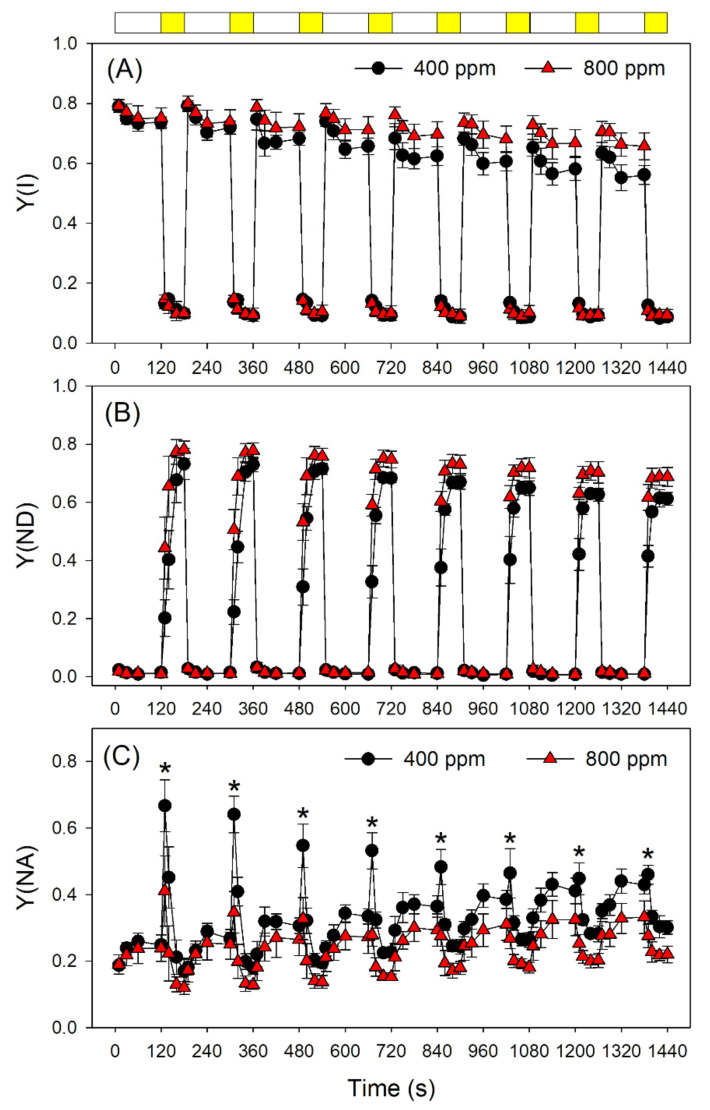
Changes in PSI parameters during fluctuating light alternating between 59 and 1809 μmol photons m^−2^ s^−1^ in Arabidopsis leaves, measured in 400 and 800 ppm CO_2_. (**A**) Y(I), the quantum yield of PSI photochemistry; (**B**) Y(ND), the quantum yield of PSI non-photochemical energy dissipation due to the donor side limitation; (**C**) Y(NA), the quantum yield of PSI non-photochemical energy dissipation due to the acceptor side limitation. Data are shown as means ± SD (*n* = 5). White bars indicate low light (59 μmol photons m^−2^ s^−1^); yellow bars indicate high light (1809 μmol photons m^−2^ s^−1^). Asterisk indicates a significant different between 400 and 800 ppm.

**Figure 4:**
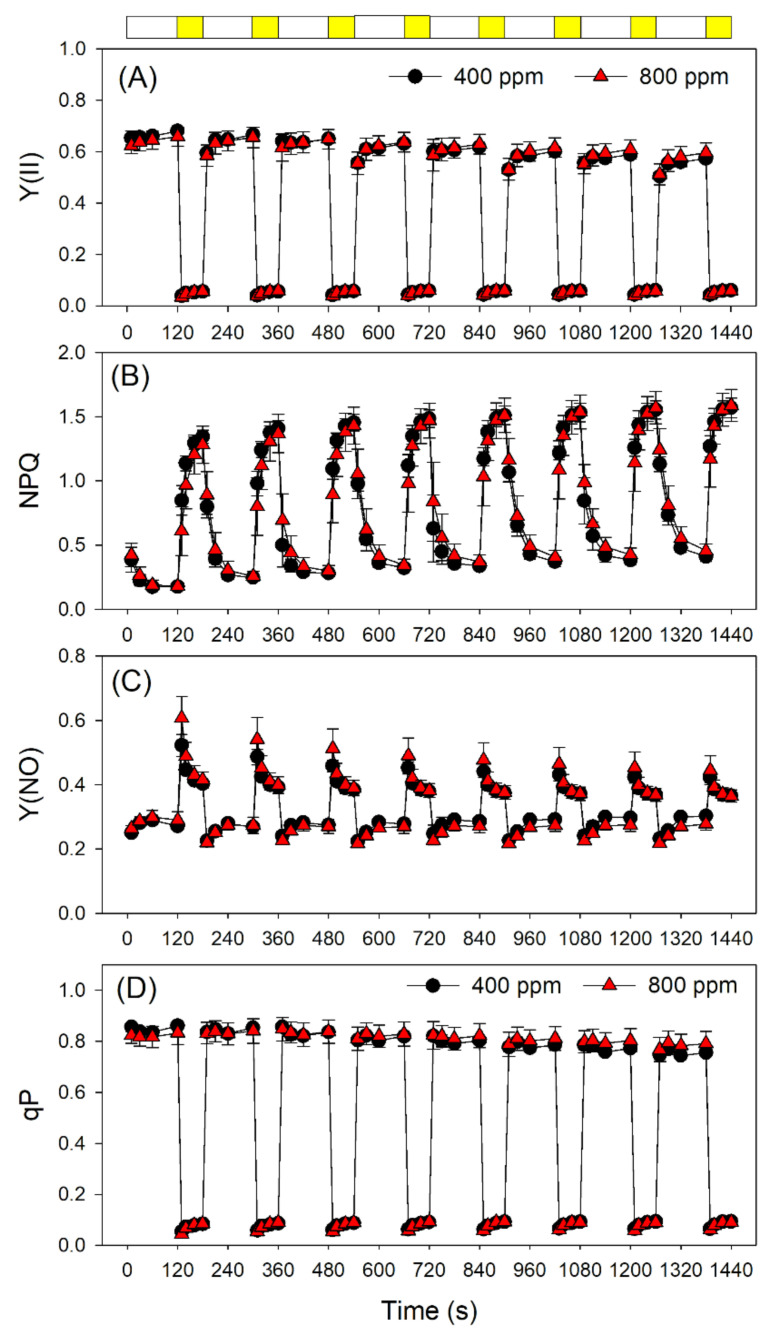
Changes in PSII parameters during fluctuating light alternating between 59 and 1809 μmol photons m^−2^ s^−1^ in Arabidopsis leaves measured in 400 and 800 ppm CO_2_. (**A**) Y(II), the effective quantum yield of PSII photochemistry; (**B**) NPQ, non-photochemical quenching in PSII; (**C**) Y(NO), the quantum yield of non-regulatory energy dissipation in PSII; (**D**) the redox state of the plastoquinone pool of PSII (qP). Data are shown as means ± SD (*n* = 5). White bars indicate low light (59 μmol photons m^−2^ s^−1^); yellow bars indicate high light (1809 μmol photons m^−2^ s^−1^).

Next, we examined the effect of elevated atmospheric CO_2_ on photosynthetic performances after transition from LL (59 μmol photons m^−2^ s^−1^) to HL (1809 μmol photons m^−2^ s^−1^). The value of quantum yield of PSI photochemistry (Y(I)) under LL was slightly higher in 800 ppm CO_2_ than in 400 ppm CO_2_ (Figure 3A). After transition from LL to HL, Y(I) did not differ between 400 and 800 ppm CO_2_ (Figure 3A). However, within the first 10 s after transition from LL to HL, Y(ND) was significantly higher in 800 ppm CO_2_ than in 400 ppm CO_2_ (Figure 3B). This rapid oxidation of PSI in 800 ppm CO_2_ alleviated the over-reduction of PSI electron carriers under FL (Figure 3C). In contrast to PSI, PSII performance under FL did not change significantly as a function of atmospheric CO_2_ concentration (Figure 4). Indeed, the effective quantum yield of PSII, Y(II), first decreased and then gradually rose upon a sudden transition from LL to HL, as expected (Figure 4A). Meanwhile, NPQ rapidly increased upon transfer to HL (Figure 4B), suggesting the gradual formation of a ΔpH. After transition from LL to HL, the quantum yield of non-regulatory energy dissipation in PSII (Y(NO)) increased sharply before undergoing a rapid drop (Figure 4C). Furthermore, the redox state of the plastoquinone pool of PSII (qP) did not differ significantly between 400 and 800 ppm (Figure 4D). 

**Figure 5:**
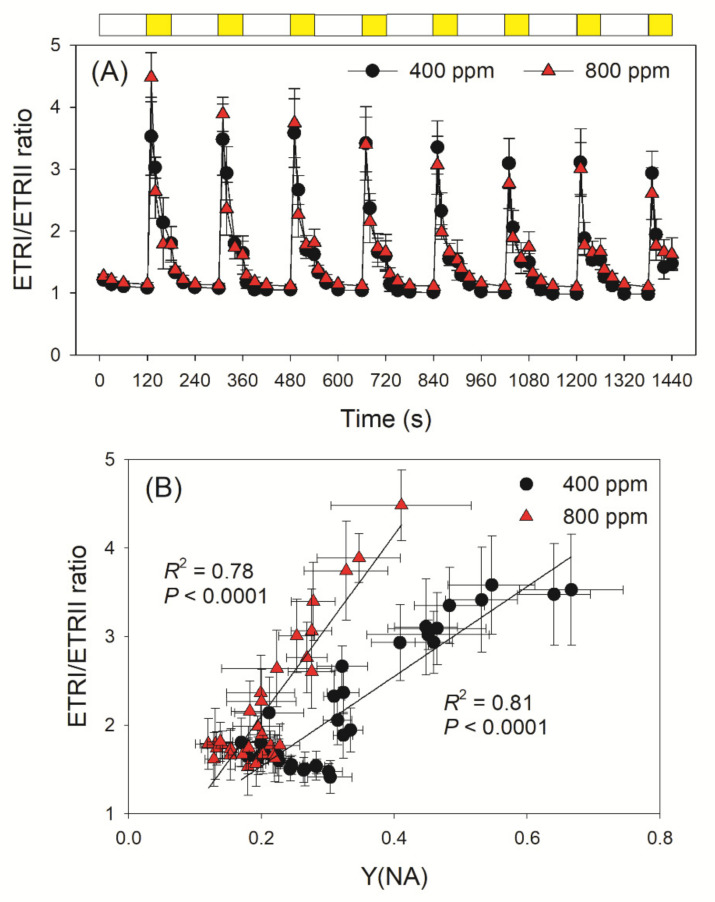
Changes in ETRI/ETRII ratio under fluctuating light and its relationship to Y(NA) in high-light phases. (**A**) Change in the ratio of ETRI/ETRII during fluctuating light alternating between 59 and 1809 μmol photons m^−2^ s^−1^ in Arabidopsis leaves measured in 400 and 800 ppm CO_2_; (**B**) Relationship between the ETRI/ETRII ratio and Y(NA) during the high light phase of fluctuating light treatments. Data are shown as means ± SD (n = 5). White bars indicate low light (59 μmol photons m^−2^ s^−1^); yellow bars indicate high light (1809 μmol photons m^−2^ s^−1^).

At LL, the ETRI/ETRII ratio was close to 1 when measured at 400 and 800 ppm (Figure 5A). Within the first 10 s after the transition from LL to HL, the ETRI/ETRII ratio was very high in exposed leaves (Figure 5A). Such an increase in the ETRI/ETRII ratio indicated that CEF was stimulated after transition to HL. Furthermore, we noticed that the change in ETRI/ETRII ratio under FL largely correlated with the PSI acceptor side limitation (Figure 5B). Once PSI was over-reduced, CEF was stimulated to help the rapid formation of ΔpH. Once the over-reduction of PSI has been relaxed, CEF activity decreased to the steady state. Therefore, CEF plays an important role in the regulation of photosynthetic rates under fluctuating light.

**Figure 6:**
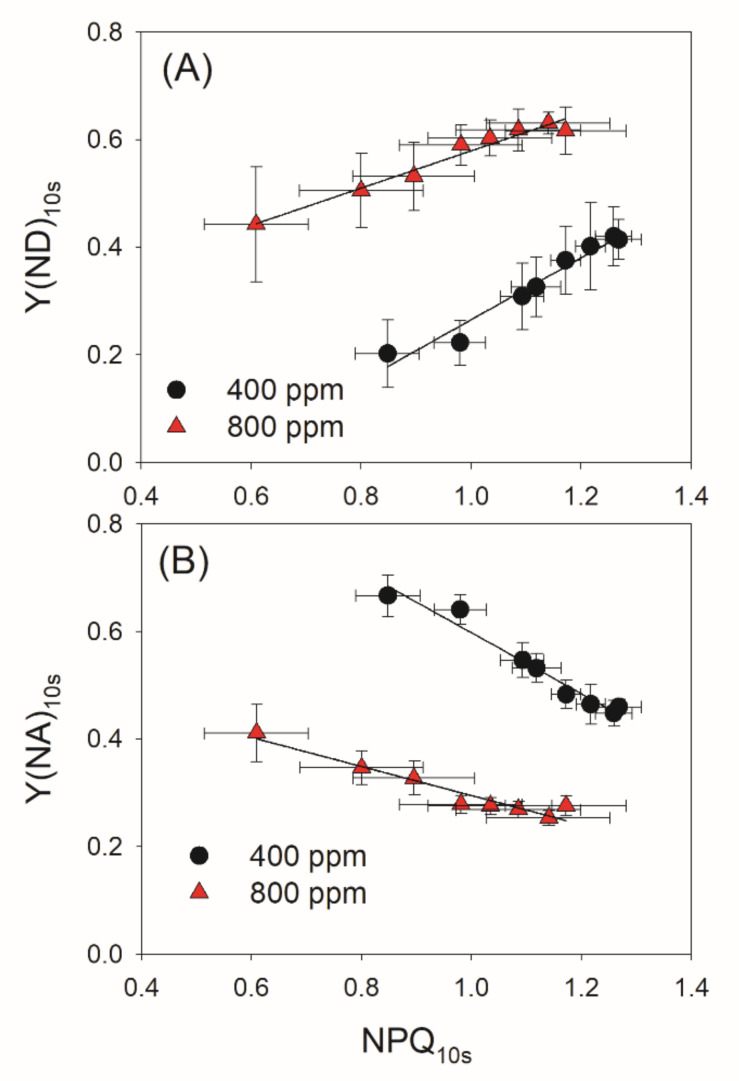
Relationships between NPQ and PSI redox state after transition from low to high light for 10 s in the eight cycles of low/high light. (**A**) Relationship between NPQ_10s_ and Y(ND)_10s_; (**B**) Relationship between NPQ_10s_ and Y(NA)_10s_. Data are prepared by combining the different time points of Figure 3 and Figure 4.

PSI redox state under FL is determined by donor- and acceptor-side regulation. To further explore the effect of elevated atmospheric CO_2_ on PSI redox state under FL, we examined the relationships between NPQ, Y(ND) and Y(NA) after transition from LL to HL for 10 s. Irrespective of the CO_2_ concentration, NPQ_10s_ was positively correlated to Y(ND)_10s_ but was negatively correlated to Y(NA)_10s_ (Figure 6). These results indicated that an increase in ΔpH facilitates the oxidation of PSI and thus prevents an over-reduction of PSI. Meanwhile, the same value of NPQ_10s_ was accompanied by a higher Y(ND)_10s_ and a lower Y(NA)_10s_ in 800 ppm CO_2_ (Figure 6), suggesting that elevated atmospheric CO_2_ affects the PSI redox state under FL mainly at acceptor side rather than at donor side.

### 3.3. Elevated Atmospheric CO_2_ Concentrations Mitigates PSI Photoinhibition under FL

**Figure 7:**
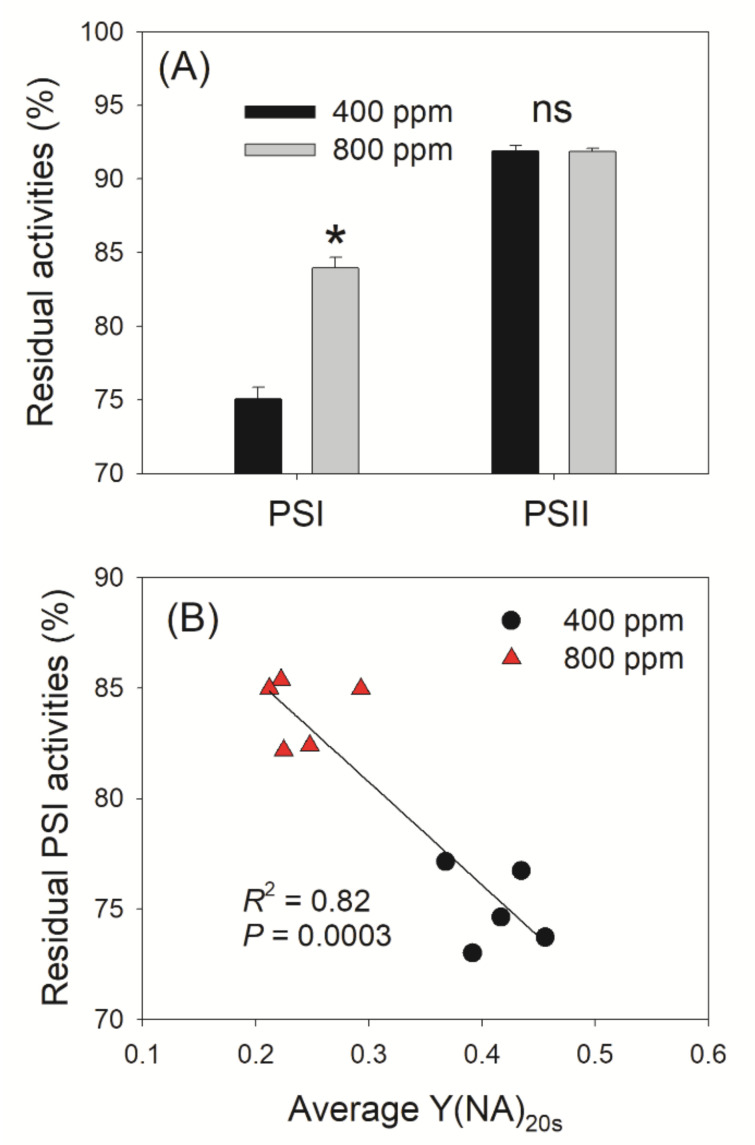
Effect of elevated CO_2_ concentration of photoinhibition under fluctuating light and its relationship to redox state of PSI. (**A**) Changes in *P*_m_ and *F_v_/F_m_* after exposure to fluctuating light for 24 min. *F_v_/F_m_* and *P*_m_ represent photosystem PSII and PSI activity, respectively; (**B**) Relationship between residual PSI activity and the mean value of Y(NA) after transition to high light for 20 s (Y(NA)_20s_). Data are shown as means ± SD (n = 5). Asterisk indicates a significant different between 400 and 800 ppm.

After FL treatment, we measured *F*_v_/*F*_m_ and *P*_m_ to evaluate PSI and PSII photoinhibition. Irrespective of the CO_2_ concentration, PSI photoinhibition under FL was more evident than that of PSII (Figure 7A). In addition, elevated atmospheric CO_2_ did not affect the extent of PSII photoinhibition but alleviated PSI photoinhibition (Figure 7A). After FL treatment, *P*_m_ decreased by 25% and 16% in 400 and 800 ppm CO_2_, respectively, relative to *P*_m_ values before FL (Figure 7A). The stronger PSI photoinhibition seen in 400 ppm CO_2_ was mainly caused by the higher Y(NA) within the first 20 s after the transition from LL to HL (Figure 7B). A higher Y(NA) represents a stronger over-reduction of PSI, which leads to the generation of ROS in PSI and thus causes PSI photoinhibition.

## 4. Discussion

Plants usually undergo FL under natural field conditions [7,14]. Following a sudden increase in illumination, the corresponding rapid rise in light absorption and electron flow from PSII cannot be immediately consumed by the CO_2_ fixation machinery, which displays much slower kinetics than ETRII [9,31,32,46]. As a result, excited states in PSI cannot be immediately transported to NADP^+^, leading to the over-reduction of PSI electron carriers and inducing the production of ROS within PSI [15,16,47]. However, ROS cannot be immediately scavenged by the antioxidant system, thereby causing photodamage to PSI [17]. A higher atmospheric CO_2_ concentration, such as that resulting from industrial activities and behind the global climate change crisis, will facilitate CO_2_ fixation after transition from LL to HL [13]. Therefore, we hypothesized that elevated atmospheric CO_2_ might mitigate PSI photoinhibition under FL.

To test this hypothesis, we measured the chlorophyll fluorescence and P700 signals under FL conditions in 400 and 800 ppm CO_2_. We documented that an elevated CO_2_ concentration significantly affected the redox state of PSI after any increase in light intensity. Upon transition from darkness to high light (1809 μmol photons m^−2^ s^−1^) for 20 s, P700 was highly reduced in 400 ppm CO_2_ but was re-oxidized in 800 ppm CO_2_ (Figure 1). Similarly, after transition from darkness to 272 μmol photons m^−2^ s^−1^ for 30 s, leaves showed a low value of Y(ND) in 400 ppm CO_2_, causing PSI to be over-reduced (Figure 2). By contrast, over the same time frame, leaves showed a high Y(ND) value in 800 ppm CO_2_, indicating that the higher CO_2_ concentration prevents PSI over-reduction (Figure 2). Furthermore, after transition from LL to HL for 20 s, the over-reduction of PSI was alleviated by the elevated CO_2_ concentration (Figure 3). When P700 is highly oxidized, the probability of electron donation from P700 to O_2_ is suppressed. Therefore, oxidation of P700 can prevent the production of ROS within PSI and thus protect PSI against excess light energy [48]. Consistently, the extent of PSI photoinhibition under FL was mainly determined by the over-reduction of PSI within the first 20 s after exposure to HL (Figure 7). Therefore, the elevated atmospheric CO_2_ concentration significantly contributed to preventing PSI photoinhibition under FL in *Arabidopsis* leaves. 

PSI photoinhibition occurs only when electrons transferred to PSI cannot be immediately transported to downstream electron acceptors [10,49,50]. The PSI redox state under FL can be regulated at the donor and acceptor sides. On the donor-side regulation, the ΔpH-dependent photosynthetic control at the Cyt *b_6_*/*f* complex slows electron transfer from PSII to PSI, thus decreasing the excitation pressure on PSI [22,24,33,51]. Upon a sudden transition from LL to HL, the transient stimulation of CEF facilitates the rapid formation of a ΔpH, which prevents uncontrolled photoinhibition of PSI under FL conditions [18,29,47]. The impairment of CEF strongly accelerates PSI photoinhibition under FL, leading to the death of *pgr5* seedlings under FL [16,18,23]. In agreement, we also observed the transient stimulation of CEF upon transition from LL to HL in both 400 and 800 ppm CO_2_ (Figure 6), indicating that CEF plays a major role in photoprotection for PSI under FL irrespective of CO_2_ concentration. In addition, either PSII photoinhibition or minimizing the activity of the oxygen-evolving complex can restrict electron flow to PSI and thus protect PSI at donor side [49,52,53]. Indeed, minimizing the activity of the oxygen-evolving complex can rescue the phenotype of *pgr5* plants under FL [52]. In this study, we observed that NPQ induction under FL is not influenced by an elevated CO_2_ concentration (Figure 4B), suggesting that higher CO_2_ levels hardly affected the formation of ΔpH under FL. Furthermore, the CEF stimulation under FL remained unchanged by the CO_2_ concentrations employed here (Figure 5). Therefore, the effect of elevated CO_2_ on the redox state of PSI under FL cannot be explained by regulation on the donor side. As shown in Figure 6, the relationships between NPQ, Y(ND) and Y(NA) under FL were altered by the elevated atmospheric CO_2_ concentration, suggesting that elevated atmospheric CO_2_ concentration influenced the PSI redox state under FL at the acceptor side rather than at the donor side.

The acceptor-side regulation of PSI is mainly attributed to electron transport from PSI to downstream electron acceptors. In photosynthetic organisms, three pathways are responsible for the regulation of PSI on the acceptor side [24,43]: (1) electron transport from PSI to NADP^+^; (2) the Mehler reaction (water-water cycle); (3) O_2_ photoreduction mediated by flavodiiron proteins (Flvs). Flvs are present in photosynthetic organisms ranging from cyanobacteria to gymnosperms but appear to have been lost in angiosperms during evolution [15,43,54]. In addition, the activity of the water–water cycle in angiosperms is species-dependent [30,45,46]. In Arabidopsis leaves, the P700 redox kinetics upon transition from darkness to light indicated that the activity of the water–water cycle is negligible (Figure 1). Therefore, the rapid oxidation of PSI under FL under elevated CO_2_ is likely to be attributed to accelerated electron transfer from PSI to NADP^+^. The electron transfer from PSI to NADP^+^ is largely affected by the NADP^+^/NADPH ratio. At elevated CO_2_ concentrations, CO_2_ fixation under FL increases [38], raising the rate of NADPH consumption. Under such conditions, the NADP^+^/NADPH ratio increases, facilitating electron transfer from PSI to NADP^+^ and thus alleviating the over-reduction of PSI electron carriers. Therefore, elevated CO_2_ affects the PSI redox state under FL through the acceptor-side regulation. 

Improving photosynthesis under FL is a critical and timely target for crop improvement under field conditions [9,13,14,55]. Maintaining high photosynthetic rates requires the avoidance of photoinhibition [6,56,57,58]. However, FL-induced photoinhibition of PSI can significantly affect CO_2_ assimilation [5]. Therefore, diminishing the extent of PSI photoinhibition under FL is an effective way to improve photosynthesis under natural FL. Raising CO_2_ concentrations can significantly boost crop yields and is thought to be attributed to enhanced photosynthetic rates [36,39]. Furthermore, we discovered here that elevated CO_2_ can significantly alter the PSI redox state under FL and thus mitigate FL-induced photoinhibition of PSI, which in turn safeguards high rates of photosynthesis. Therefore, in addition to dark reactions, elevated CO_2_ concentrations can have large effects on thylakoid reactions under FL conditions.

## Data Availability

Not applicable.
